# Teaching resources for the European Open Platform for Prescribing Education (EurOP^2^E)—a nominal group technique study

**DOI:** 10.1038/s41539-022-00141-y

**Published:** 2022-09-30

**Authors:** Michiel J. Bakkum, Bryan J. Loobeek, Milan C. Richir, Paraskevi Papaioannidou, Robert Likic, Emilio J. Sanz, Thierry Christiaens, João N. Costa, Lorena Dima, Fabrizio de Ponti, Cornelis Kramers, Jeroen van Smeden, Michiel A. van Agtmael, Jelle Tichelaar, EurOP²E consortium, Fabrizio de Ponti, Jeroen van Smeden, Michiel A. van Agtmael

**Affiliations:** 1 Amsterdam UMC, Vrije Universiteit Amsterdam, Department of Internal Medicine, Section Pharmacotherapy, De Boelelaan 1117, 1081 HV Amsterdam, Netherlands; 2 Research and Expertise Centre in Pharmacotherapy Education (RECIPE), De Boelelaan 1117, 1081 HV Amsterdam, The Netherlands; 3 European Association for Clinical Pharmacology and Therapeutics (EACPT) Education Working Group, Frankfurt, Germany; 4 Aristotle University of Thessaloniki, Faculty of Health Sciences, School of Medicine, Department of Pharmacology, Thessaloniki, Greece; 5 University of Zagreb School of Medicine and Clinical Hospital Centre Zagreb, Unit of Clinical Pharmacology, Zagreb, Croatia; 6 Universidad de La Laguna, school of Health Sciences, Tenerife, Spain and Hospital Universitario de Canarias. La Laguna, Tenerife, Spain; 7 Department of Basic and Applied Medical Sciences, Ghent University, Section Clinical Pharmacology, Ghent, Belgium; 8 Laboratory of Clinical Pharmacology and Therapeutics, Faculty of Medicine, University of Lisbon, Lisbon, Portugal; 9 Transilvania University of Brașov, Faculty of Medicine, Brașov, Romania; 10 Department of Medical and Surgical Sciences, Pharmacology Unit, Alma Mater Studiorum, University of Bologna, Bologna, Italy; 11 Department of Internal Medicine and Pharmacology-Toxicology, Radboud University Medical Center, Nijmegen, The Netherlands; 12 Division of education, Centre for Human Drug Research, Leiden, The Netherlands

**Keywords:** Neuroscience, Human behaviour

## Abstract

The European Open Platform for Prescribing Education (EurOP^2^E) seeks to improve and harmonize European clinical pharmacology and therapeutics (CPT) education by facilitating international collaboration and sharing problem-based, online, open educational resources. The COVID-19 pandemic forced teachers to switch to virtual modalities, highlighting the need for high-quality online teaching materials. The goal of this study was to establish the online problem-based teaching resources needed to sustain prescribing education during the pandemic and thereafter. A nominal group technique study was conducted with prescribing teachers from 15 European countries. Results were analyzed through thematic analysis. In four meetings, 20 teachers from 15 countries proposed and ranked 35 teaching materials. According to the participants, the most necessary problem-based-online teaching materials related to three overarching themes. Related to learning outcomes for CPT, participants proposed creating prescription scenarios, including materials focusing on background knowledge and resources on personalized medicine and topical/ethical issues such as the prescription’s impact on planetary health. Second, related to teaching, they proposed online case discussions, gamification and decision support systems. Finally, in relation to faculty development, they recommend teacher courses, a repository of reusable exam questions and harmonized formularies. Future work will aim to collaboratively produce such materials.

## Introduction

Previous studies show that final-year medical students and junior doctors throughout Europe feel insufficiently prepared to prescribe medicines safely, effectively, and responsibly^1,2^. Their lack of preparedness is reflected in the poor scores on case-based prescribing examinations and the high number of (potentially harmful) prescribing errors made in the first years after graduation^3,4^. Prescribing is a skill that is underpinned by both knowledge and attitudes. Students who have actively trained to prescribe medicines in a problem-based curriculum (i.e. with cases and simulations) are much better equipped than students who received a traditional (lecture and textbook) based training^3^. However, a recent European survey of clinical pharmacology and therapeutics (CPT) curricula found that the majority of universities still use a predominantly traditional teaching style and that transitioning to problem-based teaching is difficult^1^. In recognition of this, the international community of CPT teachers represented by the Education Working Group of the European Association of Clinical Pharmacology and Therapeutics (EACPT) has made 11 recommendations to improve and harmonize CPT education (Table 1)^1^. The European Open Platform for Prescribing Education (EurOP^2^E) was set up to specifically address recommendation 6: to utilize more online learning resources and share them nationally and internationally. From a teacher’s perspective, one of the main advantages of online learning resources is that they can be easily reused in diverse settings and locations. Previous research shows that these resources can be effective in teaching the knowledge, skills and attitudes required for safe, effective and responsible prescribing^5^. A large variety of online problem-based resources is already being used for CPT training in universities throughout Europe, but while teachers reported that they are willing to share these materials, they currently rarely do so^6,7^. Actively sharing these materials will aid local teachers to improve their CPT curricula by making them more problem-based. Moreover, the platform will allow teachers to be inspired, share teaching experiences and collaborate on new international teaching resources^6^. In light of the recent COVID-19 pandemic—which forced educators to abruptly adopt online teaching methods—the need for high-quality online teaching resources is high. Therefore, the aim of this study was to find what type of resources international CPT teachers would like to find on the platform so that these can subsequently be developed in an international collaborative manner.

**Table 1:** Recommendations of the European Association for Clinical Pharmacology and Therapeutics Education Working Group to improve and harmonize clinical pharmacology and therapeutics (CPT) education.

1	CPT should be a clear and visible programme throughout the entire medical curriculum, starting as early as possible, and should be emphasized in all clinical modules and attachments.
2	Prescribing should be trained in simulated and clinical environments, with emphasis on completing drug prescriptions, reviewing medication charts, and real responsibility for patient care.
3	Schools should formulate clear and specific learning objectives, preferably using a detailed list of core drugs (‘student formulary’) and diseases that students should be familiar with before graduation.
4	Schools should ensure that learning objectives are compatible with the learning environment and assessment activities.
5	The WHO ‘Guide to Good Prescribing’ should be used more intensively in order to teach and train rational prescribing.
6	Schools should utilize more online learning resources and preferably share these at the national or international level.
7	Medical/pharmacy students and junior doctors should be engaged in ‘near peer’ education, supervised and trained by clinical pharmacologists and senior clinicians.
8	Clinical pharmacists and nurse prescribers should be given a greater role in the development and delivery of CPT education.
9	Schools should implement a robust and separate CPT assessment structure throughout the curriculum, with no compensatory mechanism (i.e. the possibility to get a sufficient score based on other subjects).
10	Schools should implement a valid and reliable final prescribing assessment at or near the end of the medical curriculum to assess whether graduates are able to prescribe safely and effectively.
11	Prescribing should be assessed in a simulated or clinical context, with emphasis on writing prescriptions, verifying the suitability of the treatment choice, giving information to patients, and drug monitoring.

These recommendations were previously published by Brinkman et al.^1^ (CC BY-NC-ND 4.0).

## Results

Sufficient data were collected by the fourth scheduled meeting (no new suggestions were made in this meeting). In total, 20 CPT teachers from 20 institutions in 15 countries participated. Two additional teachers provided informed consent but did not participate (one was a “no show” for the first meeting, and one could not attend any of the four meetings). The meetings lasted 85–110 min.

### Ranking results

Table 2 shows the final rankings of the four meetings.

**Table 2:** Nominal group results.

(a) First meeting (five participants from Ireland, Malta, Netherlands, Poland, and Romania)			
Ranking	The participants suggested to include:	Average score (out of 5)	Number of votes
1	Training teachers in problem-based learning	3	4
2	Prescribing scenarios enriched with real patient data	2.6	4
3	Virtual interactive patients	2	3
4	Database of exam questions	1.8	4
5	A collection of what is new in pharmacotherapy education	1.6	3
6	Prescribing scenario about pharmacogenomics	1.6	2
7	Teacher community (discussion platform)	1	3
8	Transdisciplinary education between MD/pharm students	0.4	1
9	Role-playing clinical cases	0.2	1

(b) Second meeting (five participants from Belgium, Croatia, Estonia, Netherlands, and Serbia)			
Ranking	The participants suggested to include:	Average score (out of 5)	Number of votes
1	International online debate	3.6	5
2	Prescribing games	3.4	5
3	Clinical case repository with background information	3	5
4	Case-based therapeutic reasoning	2.6	4
5	Polypharmacy tool	1	3
6	Tool about medication safety in pregnancy/lactation	0.8	1
7	Prescribing scenarios on drug allergies	0.4	1
8	Adverse drug reaction tool	0.2	1

(c) Third meeting (four participants, two from UK, one from Finland, and one from Spain)			
Ranking	The participants suggested to include:	Average score (out of 5)	Number of votes
1	Realistic interactive cases	3.75	3
2	Practicality of prescribing	2.5	4
3	Topical societal issues (“not in the textbook stuff”)	2.25	3
4	Interprofessional problem solving	1.75	2
5	Database of exam questions	1.5	3
6	Interactive digital resource on medicine regulations, drug discovery, and ethics	1.25	1
7	Task on clinical situations where there is low/no evidence	1	1
8	“Meta-competences” in prescribing	0.75	2
9	Resource on rapidly evolving areas	0.25	1

(d) Fourth meeting (six participants, from Denmark, Finland, Germany, Italy, Netherlands, and Spain)			
Ranking	The participants suggested to include:	Average score^a^ (out of 5)	Number of votes
1	Repository of clinical cases	4.0	6
2	Knowledge multimedia—Clips, Images, podcast sessions	3.2	6
3	Adaptive e-modules on longitudinal cases.	2.5	4
4	Prescribing scenarios including clinical decision support	2.3	4
5	Personalized formularies	1.2	3
6	Database of exam questions	1.0	4
7	Slides/videos on topical issues	0.7	2

^a^One participant provided a top four instead of top five.

### Thematic analysis

**Fig. 1:**
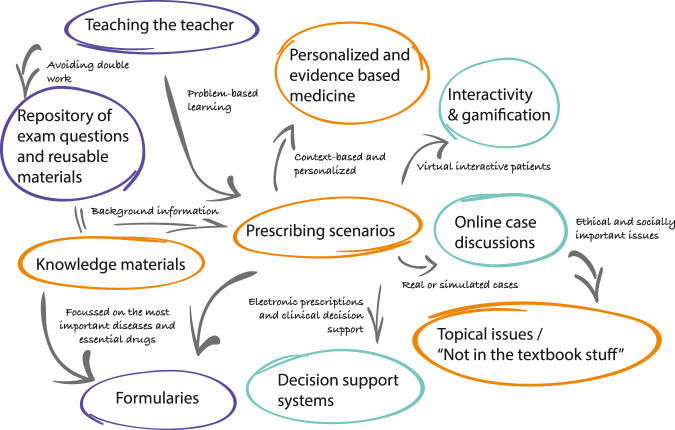
Visual representation of the thematic analysis. Orange = related to learning outcomes for Clinical Pharmacology and Therapeutics; Cyan = related to format of teaching; purple = related to resource and faculty development.

The suggestions of the participants fitted into ten themes which were prioritized according to the ranking results (Table 3). They related to three overarching themes: learning outcomes for CPT, the format of teaching and resource and faculty development. Figure 1 presents an overview of these themes and the relationships between them.

**Table 3:** Overview and prioritization of the identified themes.

Priority	Theme
1	Prescribing scenarios
2	Interactivity and gamification
3	Repository of exam questions and reusable materials
4	Online case discussions
5	Decision support systems
6	Teaching the teacher
7	Knowledge materials
8	Topical issues / “not in textbook stuff”
9	Personalized and evidence-based medicine
10	Formularies

#### Themes related to learning outcomes for CPT

##### Prescribing scenarios

Prescribing scenarios for clinical cases were ranked as being the most important. According to the participants, these cases should focus on prescribing essential drugs for the most common diseases and be aligned to the student level, ranging from patients with single health problems for first-year students to more real-life patients with multiple health problems for advanced students, including training in a medication review and deprescribing. Besides deprescribing, three other specific subjects were suggested, namely, pharmacogenomics, drug allergies, and rapidly evolving areas such as biologicals. Since diagnostic and therapeutic reasoning are often not linked in CPT cases, participants proposed they be combined, so that students also learn to interpret clinical data, such as physical examination findings, laboratory results, and radiological findings. Participants suggested that these cases could be presented in the form of pre-recorded videos of clinical consultations, role-playing simulations in which the students alternate between playing the doctor and the patient, live online case discussions, and interactive gamified virtual patients. To emphasize how prescribing is context-based and should be aimed at the personal needs of the patients, the participants proposed (in two of the meetings) making adaptive and/or longitudinal cases where the patients’ situations change or their health problems progress.

##### Knowledge materials

Participants suggested creating (video) clips and podcasts about thematic issues that—other than most prescribing scenarios—should focus on the common denominators of clinical pharmacology (i.e. that do not differ per country), such as prescribing for special populations, renal function, and deprescribing. It was acknowledged that it would be challenging to condense 2-hour lectures into clips of maximally 10–12 min. Videos of the working mechanisms of different classes of drugs, rather than of specific drugs, would also improve international generalizability. The participants suggested that these materials be used as a pre-class activity in flip-the-classroom style lessons.

##### Topical issues (“Not in textbook stuff”)

A suggestion made in two meetings was to cover “important aspects of prescribing that you will not find in textbooks” [participant from UK(1)], such as socially important, topical, and more ethical issues (antimicrobial resistance, the opioid pandemic, environmentally sustainable prescribing or “ecopharmacostewardship”, and inequality in medicine). These topics, or “attitudes underpinned by knowledge” [participant from UK(2)], are difficult to teach but vitally important. Generating international standpoints on these matters may help teachers to integrate them into their local curricula. A specific example of a topical issue was the interaction with the pharmaceutical industry. Participants argued that students and doctors are insufficiently exposed to the processes of drug discovery, drug development, and medicines regulation and marketing, and an appreciation of these topics would probably influence how doctors prescribe and appraise potential conflicts of interest.

##### Personalized and evidence-based medicine

Although it could be classified as *“*not in textbook stuff*”*, the need to pay more attention to personalized medicine was mentioned separately, sometimes in the context of prescribing scenarios. According to one participant [UK(1)], students need to appreciate the nuance between prescribing as science and prescribing as art and should be taught to “challenge the heuristics” and learn when not to trust or apply evidence-based guidelines. This participant explained, “It drives me mad when a 95-year-old bedbound patient is put on 80 mg of atorvastatin just because she had a mild troponin rise”. It was further noted that “medical students have no idea of the reimbursement of medicines” [participant from Italy], and that this is a very practical and important point to consider in personalized prescribing. Lastly, students should be taught how to appraise the literature in situations where there is no or limited evidence available, such as treatment for COVID-19 at the start of the pandemic.

#### Themes related to the format of teaching

##### Interactivity and gamification

Gamification was mentioned, both as a separate resource and in the context of prescribing scenarios. Participants suggested that interactive virtual patients could be created that respond realistically to different treatments or management strategies, thereby giving students (instant) feedback and making it possible for them to assess their treatment choices. This could be especially useful for acute medical situations that students may otherwise not be exposed to. A time limit could be imposed to create a sense of urgency. One participant was already using gamification, whereby students progressed through a patient case by opening lockers, as in an escape room. This participant found that gamification helped keep students engaged with what was being taught. Other forms of gamification mentioned were short quizzes with in-class competition (like Kahoot!, www.kahoot.it) and a suggestion for a drug–drug interactions rehearsing game.

##### Online transdisciplinary, transnational case discussions

The participants suggested holding online discussions of (real or simulated) cases, which would enable students from different health professions to collaborate in real-time, much like they will do in their future professions. Additionally, online discussions would allow students to identify and discuss differences in prescribing guidelines and attitudes between countries, so that they could learn that “in many situations, there is no such thing as one right answer” [participant from Belgium]. Participants suggested that these meetings be held via live videoconferencing, with students rather than teachers taking the lead. These sessions could be recorded for later reference. The discussions should include ethical aspects such as planetary health and other problems not found in textbooks. The platform would function as a meeting place and catalyst for teachers who wish to organize such meetings.

##### Decision support systems

Participants suggested using (clinical decision) support and electronic prescribing systems for educational purposes. Medical students appear to be more comfortable with the therapeutic decision-making process than with the practical aspects of prescribing, and participants suggested that students should be trained “in the scribing bit of prescribing” [participant from UK(1)], using electronic prescribing systems. Ideally, these systems should show realistic decision support alerts so that students learn to react to prescribing red flags. Several participants were already training students in the sandbox environment of their electronic patient files and stated that making such a resource available for international use would have to account for local/national differences in these systems. Other suggestions included standalone polypharmacy tools, such as an interaction checker. As pointed out, these systems already exist and are helpful in teaching students about drug–drug interaction and how to interpret alerts. The participants also suggested creating similar tools regarding the safety of medication during pregnancy and lactation and identifying adverse drug reactions on the basis of patient symptoms.

#### Themes related to resource and faculty development

##### Repository of exam questions and other reusable materials

RParticipants suggested keeping existing teaching materials in online repositories so that they can directly re-use them in their own teaching or use them as a source of inspiration. They suggested collecting knowledge-oriented and case-based exam questions (including rubrics), slides of lectures, videos and figures. Additionally, because “most students only like to learn the minimum of minimum” [Participant from Serbia], participants proposed collecting web links to further reading materials for the more interested students.

##### Teaching the teachers

The top-ranking suggestion in the first meeting was for so-called teach-the-teacher materials. In the other meetings, this resource was discussed in relation to international differences and the digital readiness of teachers. Teaching teachers how to use problem-based learning was considered most important because there are relatively few teachers, and problem-based learning is more time intensive (and therefore costly) than traditional teaching. Moreover, teachers are often unwilling to change their way of teaching, because “they think they know it all” [participant from Malta] and are too busy to do so. Participants who experienced a shortage of teaching colleagues agreed that an international teach-the-teacher course on problem-based learning may help to attract more teachers or enable pharmacists and other paramedical professionals to qualify to teach CPT. Keeping up-to-date with the newest teaching innovations was considered essential, and the platform should keep an overview of new innovations. Additionally, participants would like a forum or Twitter-like discussion board so that they could ask each other for help.

##### Formularies

Lastly, participants discussed the need to focus our efforts on the most common diseases and/or the most commonly prescribed drugs. A European reference should be established for the most important drugs that medical students should know about. The participants believed that such a list would easily contain 200–300 drugs, which they deemed too many. Therefore, they suggested including ways to extract information into personalized formularies with fewer drugs. The Spanish and English P-drugs app (and website) already exist, and this app could be easily translated and made available to other countries.

## Discussion

This study marks an important step in improving and harmonizing CPT education. It provides a clear and prioritized overview of the teaching resources European CPT teachers need and helps the community with practical ideas for the creation of these resources. The open (free to re-use, adapt, and redistribute) distribution of these resources on EurOP^2^E will likely help to make high-quality problem-based CPT education accessible for all.

A comparison of our findings with those of an earlier overview of digital educational resources used for prescribing education^7^ shows that many of the proposed resources already exist in a similar form. However, with few exemptions (e.g. a repository of pharmacological illustrations, which is available as the teaching resource centre via http://trc-p.nl), such resources are restricted to local universities and not known or available to the participants. This shows how little CPT educators currently collaborate and emphasizes that EurOP^2^E should not only be about creating new resources but also about making existing ones openly available. The list of suggestions will probably change once CPT educators have become more accustomed to collaborating and sharing materials, and EurOP^2^E will have to be dynamic towards this.

When we compare the learning outcomes mentioned in this study to the previously established list of key learning outcomes for CPT education published in 2017^8^, we see a remarkable new interest in overprescribing and the impact of pharmaceuticals on planetary health (as well as much emphasis on the potentially mitigating effects of non-pharmacological interventions and deprescribing). The Association for Medical Education in Europe (AMEE) has recently published a global, collaborative, representative, and inclusive vision on how to educate an interprofessional workforce that can provide sustainable healthcare and promote planetary health^9^. The Association recommends improving faculty engagement and development^9,10^. Therefore, we suggest that standpoints are established collaboratively, and teach-the-teacher materials and (templates for) specific lessons are developed and then shared via EurOP^2^E^11^. A similar approach may be viable for other topical/ethical issues that were mentioned in this study, such as race-based medicine^12^, gender inequality in the medical literature, and working with the pharmaceutical industry.

The COVID-19 pandemic forced medical educators to abruptly switch to online teaching. While this has brought challenges, such as student engagement and focus, the pandemic has also been described as the “long-awaited and much-needed catalyst for a new online teaching era in medical education”^13^. This silver lining is particularly apparent for interprofessional education, partly because of positive experiences gained in the interprofessional anti-COVID approach^14^, but also because experience with online education has made it much easier to bring students (and healthcare workers) of different professions together. The goal of interprofessional education is to learn with, from, and about each other to improve collaboration and the quality of care^15^. For prescribing education, this usually means pharmacy students and medical (and/or non-medical prescribing) students learning together^16,17^. Although the participants suggested facilitating this type of interprofessional education via EurOP^2^E, they also thought that contact among international students would lead to an understanding/awareness of international differences in guidelines, medicine regulations, and prescribing attitudes. These very differences have previously been described as major barriers to international collaboration^6^. While these differences may indeed reduce the applicability of existing resources and exam questions, this could be overcome by making the teaching materials adaptable and/or aimed at common elements of prescribing education (e.g. pharmacokinetics and dynamics). Moreover, identifying and discussing international differences may raise awareness of context-based medicine and that there is not necessarily one correct prescribing solution.

Providing learning experiences for teachers was another theme identified in this study. The overall goal of EurOP^2^E is to help teachers improve their teaching practice. While this may in itself be viewed as a teach-the-teacher activity, the results of this study have helped us realize that the actual teaching of teachers should be viewed as one of the means to this goal. Unfortunately, it can be a challenge to motivate professionals to adopt new techniques/methods and therefore, attention should be paid to good practice in faculty development^18,19^. Unlike institutional faculty development programmes, which often use external motivators (i.e. promotion on the academic ladder), EurOP^2^E will have to appeal to the intrinsic motivation of teachers. Being mindful of the principles of the self-determination theory may help to do so^20,21^. However, the participants also thought that high-quality teach-the-teacher courses would help to attract new CPT teachers, but this remains to be seen. Depending on the need, teach-the-teacher courses could cover generic skills and learning theories, such as courses on problem-based learning and what is new in CPT education or about more specific topics.

This study had some limitations. First, about half of the sent-out invitations to participate remained unanswered, and because of that, we did not include participants from some of the larger EU member states (i.e. France, Portugal and Hungary). However, we believe this is not a problem because we gathered sufficient data from the other countries and have no reason to assume intercountry differences. Moreover, we view the results as a starting point to a dynamic list of resources to create, and new suggestions remain welcome. Secondly, because the participants were all busy professionals, we thought that a time investment of ~90 min was the most we could ask of them. In retrospect, this was a bit short because, in three of the four meetings, we had to stop the second phase before all suggestions had been made and slightly rushed the third phase. This was adequately handled by allowing the participants to add all suggestions that they felt were crucial. Because we continued interviewing groups until no new suggestions came to light, we are confident that we captured all relevant ideas. Thirdly, we noted before that not all suggestions were truly new, and we recognize that this may be due to priming. For example, many of the participants had also participated in a Delphi study aimed at developing a European list of essential medicines^22^, which may explain how this came to be a theme in this study.

In conclusion, the most urgently needed online problem-based educational resources for clinical pharmacology and therapeutics related to the learning outcomes, the format of teaching and resource and faculty development. Depending on the theme, the function of EurOP^2^E will vary from supporting and facilitating international communication and collaboration to providing teach-the-teacher materials and/or (initiating) the collaborative production of ready-to-use teaching materials. We identified the planetary health impact of prescribing as a new learning outcome for clinical pharmacology and therapeutics education.

## Methods

This study used the nominal group technique (NGT) combined with a thematic analysis of the discussions. NGT is a consensus-building technique wherein participants have an equal and uninterrupted opportunity to present their expert opinions and ideas to the group^23^. After all, participants have presented their ideas, the group then discusses, alters, scraps, or combines ideas. Thereafter, the participants independently and anonymously rank these ideas. We chose this method because it leads to a clearly prioritized list of suggestions and prevents certain more vocal participants from promoting their ideas or dominating the meeting, and, compared with other consensus methods (e.g. Delphi), enables participants to present their ideas in detail^23^. We additionally performed a thematic analysis of the group discussions. This allowed us to gain a more conceptual understanding of the nominal results and thus identify commonalities between the individual group discussions. The original ranking results helped us in prioritizing the identified themes.

### Study participants

Members of the Network of Teachers in Pharmacotherapy education (NOTIP), which consists of 400+ teachers in pharmacology and CPT from all EU countries, United Kingdom, Norway, and Serbia, were asked to participate in the study. We used purposive sampling whereby one or two NOTIP members per country were selected on the premise that they were active in teaching innovation and probably motivated to participate, and/or had participated in previous research studies. In total, 39 participants were asked to participate via e-mail. Invitees were free to forward the e-mail to one or more colleagues if they deemed them to be more qualified to participate. Non-responders received one reminder after 2 weeks.

### Data collection

Four meetings were scheduled for October 2021, with the possibility to have additional meetings if insufficient data were collected. The meetings were held online via Microsoft Teams in groups of 4–6 participants. After a brief presentation on the study aims and a round of introductions, the interviews continued in four phases: (1) Participants silently and privately organized their thoughts for 5 min; (2) One by one (in a round-Robin fashion), participants presented their ideas; (3) group discussions were held with a view to clarifying and combining the ideas from step 2; (4) The participants anonymously voted for their top-5 of remaining ideas. For the second phase, we continued until all participants ran out of ideas, or until (after a minimum of three full rounds) time demanded that we continued to the next phase, in which case all participants were given the last opportunity to present any crucial ideas. The meeting host recorded all ideas on a virtual flip-over (google Jamboard, http://jamboard.google.com), which was visible to all participants via screen-sharing. For the voting-phase, we used Mentimeter’s (http://mentimeter.com) multi-voting question type. To make the overall ranking, a participant’s first choice was awarded 5 points, second choice 4 points etc. We concluded the meetings by sharing this overall ranking with the participants.

Three researchers were present for all meetings: MB was the host; BL provided technical assistance to participants and prepared and launched the voting system; JT observed, kept time, and double-checked the host (intervened when necessary). Right after each meeting, the researchers discussed points of improvement for the next meetings and whether sufficient data had been collected. All meetings were audio and video recorded using Microsoft teams.

### Data analysis

The voting results for the individual suggestions are presented as the average score per participant (sum of scores/number of participants) and ranked accordingly. When there was a tie, the number of participants that voted for a given a suggestion decided the ranking. Additionally, we transcribed the recordings of the meetings verbatim and used a thematic analysis^24^, whereby BL and MB together (through repeated reading of the transcripts and discussion) developed a final set of codes and initial themes in MAXQDA (standard 2020). Using these codes, they recoded all transcripts and independently reviewed the themes. In a meeting together with JT, they finalized and named the themes. Lastly, the remaining authors, who are CPT teachers, provided feedback on the results. Six of the authors had also participated in the meetings, thus for them, this was a check of internal validity (member check). For the other five authors, it was an external validation of the results. The consolidated criteria for reporting qualitative research (COREQ) checklist^25^ guided the reporting of this study.

### Reflexivity

MB is a doctor and PhD student in the EurOP^2^E project, with 4 years of experience in research in CPT education and teaching pharmacotherapy. JT is a PhD-grade associate professor in pharmacotherapy with over 17 years of experience as a teacher and coordinator of pharmacotherapy education and research in CPT education. BL is a medical student and student-teacher in pharmacotherapy who joined the research team for his master’s thesis. All other authors are (associate) professors in clinical pharmacology and part of the international EurOP^2^E consortium. The research team had a constructivist approach^26^ to the thematic analysis and viewed the results in light of the WHO Guide to Good Prescribing’s Six-step method for problem-based pharmacotherapy education^27^ and the framework for the EuroP^2^E platform^6^.

## Data Availability

The datasets generated during and/or analyzed during the current study are available in the Open Science Framework repository, https://osf.io/62u8a/^28^.
